# *ZNF496* as Candidate Gene for Neurodevelopmental Disorders: Identification of a Pathogenic De Novo Frameshift Variant

**DOI:** 10.3390/ijms26157586

**Published:** 2025-08-05

**Authors:** Francesco Calì, Miriam Virgillito, Simone Treccarichi, Antonino Musumeci, Pinella Failla, Carla Papa, Rosanna Galati Rando, Concetta Federico, Salvatore Saccone, Mirella Vinci

**Affiliations:** 1 Oasi Research Institute-IRCCS, 94018 Troina, Italy; cali@oasi.en.it (F.C.); streccarichi@oasi.en.it (S.T.); amusumeci@oasi.en.it (A.M.); pfailla@oasi.en.it (P.F.); cpapa@oasi.en.it (C.P.); rgalati@oasi.en.it (R.G.R.); 2 Department Biological, Geological and Environmental Sciences, University of Catania, Via Androne 81, 95124 Catania, Italy; virgillito.miriam@gmail.com (M.V.); concetta.federico@unict.it (C.F.)

**Keywords:** zinc finger proteins, *ZNF496*, neurodevelopmental disorders, KRAB domain, C2H2 zinc finger domain, *JARID2*

## Abstract

Zinc finger proteins are frequently implicated in a wide range of neurodevelopmental disorders (NDDs). In this study, we report a case of mild intellectual disability (ID), global developmental delay (GDD), and developmental coordination disorder (DCD) in an individual with unaffected parents. Trio-based whole-exome sequencing (WES) identified a de novo variant (c.1530dup, p.Glu511ArgfsTer16) in the *ZNF496* gene of the proband. According to ACMG guidelines, this novel variant is classified as pathogenic. It creates a frameshift that introduces a premature stop codon, resulting in a truncated protein of 525 amino acids (compared to the wild-type 587 residues). Notably, NMDEscPredictor analysis predicted that the transcript escapes nonsense-mediated decay (NMD) despite the frameshift. Computational analyses suggest the potential pathogenetic effects of the identified variant. As documented, ZNF496 interacts with *JARID2*, a gene associated with NDDs, ID and facial dysmorphism (MIM: #620098). In silico analyses suggest that the identified mutation disrupts this interaction by deleting ZNF496’s C2H2 domain, potentially dysregulating *JARID2* target genes. To our knowledge, this is the first reported association between *ZNF496* and NDDs, and the variant has been submitted to the ClinVar database (SCV006100880). Functional studies are imperative to validate *ZNF496*’s role in NDDs and confirm the mutation’s impact on ZNF496-JARID2 interactions.

## 1. Introduction

Zinc finger proteins belong to a superfamily characterized by domains that specifically bind DNA [1,2]. These proteins play key roles in various mechanisms of gene regulation. Their regulatory functions are mediated by binding to RNA or DNA, and zinc finger proteins have been implicated at multiple levels of regulation, including splicing, capping, polyadenylation, export, localization, translation, and decay [3,4]. Consequently, it is likely that many genes are regulated by zinc finger proteins. This hypothesis underscores the importance of zinc finger proteins (ZNFs) in human brain development, contributing to its complexity [5].

As previously reported, zinc finger (ZNF) proteins are significantly associated with the pathogenesis of neurodevelopmental delay. The typical zinc finger protein of the C2H2 class is characterized by two conserved cysteine residues, two conserved histidine residues, and a zinc ion, which together form two *β*-sheets and one *α*-helix. Numerous C2H2-ZNF proteins—representing approximately 10% of human proteins—are involved in key neuronal processes such as cell coordination, proliferation, differentiation, migration, and apoptosis. They regulate specific neuronal functions, and alterations in C2H2-ZNF proteins may contribute to both morphological and functional anomalies.

C2H2-ZNF proteins containing KRAB and SCAN domains have been implicated in neurodevelopmental delay, a condition typically characterized by impairments in higher brain functions, including cognitive deficits, memory loss, and emotional dysregulation [6,7]. A broad array of genes encoding KRAB zinc finger proteins has been associated with neurodevelopmental disorders (NDDs). Haploinsufficiency of four KRAB-ZNF genes—*ZNF302*, *ZNF181*, *ZNF599*, and *ZNF30*—has been proposed as the underlying cause of 19q13.11 microdeletion syndrome, which is characterized by neurodevelopmental delay [8]. Additionally, biallelic variants in *ZNF142* have been linked to a syndromic form of neurodevelopmental disorder [9]. These alterations are observed in a range of disorders such as intellectual disability (ID), autism spectrum disorder (ASD), schizophrenia, major depressive disorder (MDD), and bipolar disorder (BD) [10,11]. Although C2H2-ZNF proteins are present across many organisms, KRAB-ZNF and SCAN-ZNF proteins have emerged exclusively in vertebrates and mammals, highlighting their evolutionary adaptation to support complex functions in higher organisms [5,7]. High levels of KRAB-ZNF expression have been detected in several neurodevelopmental disorders, particularly in ID, ASD, and other psychotic conditions associated with dysfunctions of higher-order brain processes. Moreover, defective poly-zinc finger proteins have been linked to additional cerebral malformations, including microcephaly, hypoplasia, and other localized anomalies [11].

Among KRAB-ZNF and SCAN-ZNF zinc finger proteins, particular emphasis has been placed on ZNF496 (formerly known as NIZP1), which stands out as a key protein involved in DNA, RNA, and histone binding activities [12,13].

In this study, WES analysis identified the de novo variant c.1530dup in the *ZNF496* gene in a patient affected by mild ID, GDD, and DCD with unaffected parents.

The purpose of this work is to associate the *ZNF496* gene with the neurodevelopmental disorder observed in the patient’s clinical examination.

## 2. Results

### 2.1. Clinical Report

The 10-year-old girl is the first daughter of healthy, non-consanguineous parents. During pregnancy, at the 31st week, a decrease in the growth curve was observed, followed by fetal growth arrest. She was delivered by cesarean section at 34 weeks of gestation. The birth weight was 1850 g, which is below the normal range, while the head circumference at birth was 31 cm.

She was mechanically ventilated for 3 days. No abnormalities were present on transfontanellar ultrasound. She showed patency of the foramen ovale.

She walked independently at 14 months, first words at 12 months, and sphincter control at 4 years. The first signs of developmental alteration appeared around 18 months of age, with regression of some acquired skills and the onset of social withdrawal and stereotyped behaviors. The phenotype did not show evident dysmorphic features; however, her head circumference was smaller than expected for her height, suggesting a possible case of relative microcephaly. Current anthropometric measurements are height 142 cm (50° perc.), and weight 35 kg (50° perc.), OFC: 50 cm (2° perc.).

She has mild intellectual disability. Behaviorally, she shows a limited attention span but appears cooperative and demonstrates a fair level of autonomy in daily activities.

Her semantic lexical knowledge, assessed by the Peabody test, is poor, and her verbal production consists of complete sentences that are not always coherent with the communicative context.

The patient underwent further genetic analyses, including standard karyotyping, CGH-array, and *FMR1* gene testing, all of which returned normal results.

### 2.2. WES Analysis

WES trio revealed the presence of a heterozygous de novo nucleotide variation at position c.1530dup in the *ZNF496* gene (NM_032752.3). WES analysis unveiled no additional mutations directly or potentially associated with neurodevelopmental disorders. This suggests that the identified mutation could be responsible for the clinical phenotype.

The variant was classified as photogenetic according to ACMG criteria PVS1, PM2, and PS2, as described in Table 1.

**Figure 1:**
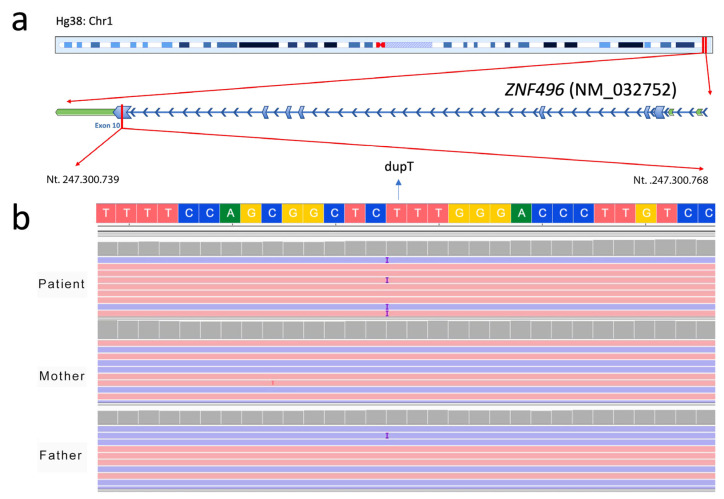
Identification of the c.1530dup genetic variant in the *ZNF496* gene (NM_032752.3) by NGS. (**a**) Schematic representation of the chromosomal localization of *ZNF496*, highlighting the position of the identified variant within exon 10 of the gene. (**b**) Visualization of the de novo insertion of a thymine (“T”) at position 1530 (c.1530dup) in the proband (dupT), which is absent in both unaffected parents. The image was obtained using the Integrative Genomics Viewer (IGV) software version 2.19.5.

Figure 1 shows the chromosomal localization of the *ZNF496* gene (Figure 1a) and presents the NGS output analysis for the identification of the c.1530dup genetic variant (Figure 1b).

**Figure 2:**
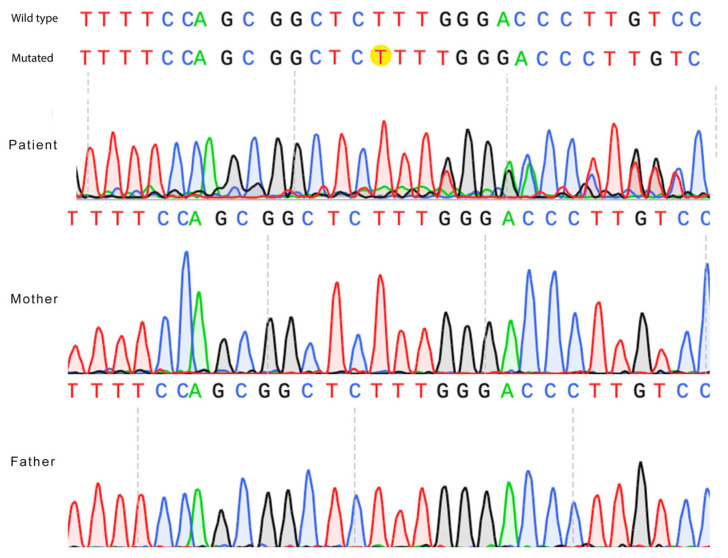
Confirmation of the *ZNF49* c.1530dup de novo variant by Sanger sequencing. Electropherograms of the proband and both parents are shown. The thymine nucleotide insertion (c.1530dup) (“T” nucleotide) is highlighted in yellow in the proband’s sequence (mutated) and is absent in the parental sequences (wild type).

The thymine duplication in position 1530 causes a frameshift mutation and alters the reading frame from this point. Sanger sequencing has confirmed the de novo variant (Figure 2).

The DOMINO prediction tool suggests an uncertain inheritance pattern of the *ZNF496* gene with a score of 0.582 (scale 0–1).

The bioinformatic tool NMDpredictor predicts that the variant located at position 1530 of the NM_032752.3 transcript of *ZNF496* escapes nonsense-mediated decay (NMD).

### 2.3. Structural Protein Prediction of ZNF496

The ZNF496 protein is composed of 587 residues, which include disordered regions and functional domains annotated in ProRule. The mutation p.Glu511ArgfsTer16 is located within a disordered region of the Zinc Finger C2H2 domain (IPR013087).

MuPro analysis underlines that the variant decreases protein stability, as indicated by the delta delta G score of −0.747 (score range: −1 to 1). The formation of a premature stop codon causes alteration of the polypeptide chain and leads to the formation of a mutated protein containing only 525 amino acids, compared to the wild-type protein, which contains 587. Analysis through AlphaFold3 generated 5 structural models for each protein prediction done for both the wild-type and the mutated ZNF496 proteins. Notable structural differences between the wild-type and mutated proteins were observed. The wild-type predicted models contain an average of 241.4 (±3.56) hydrogen bonds, while the mutated protein models contain 191 (±6.12) hydrogen bonds.

For subsequent analyses, the best model was selected based on the highest predicted local distance difference test (pLDDT) score, as described in Section 4 (Materials and Methods).

**Figure 3:**
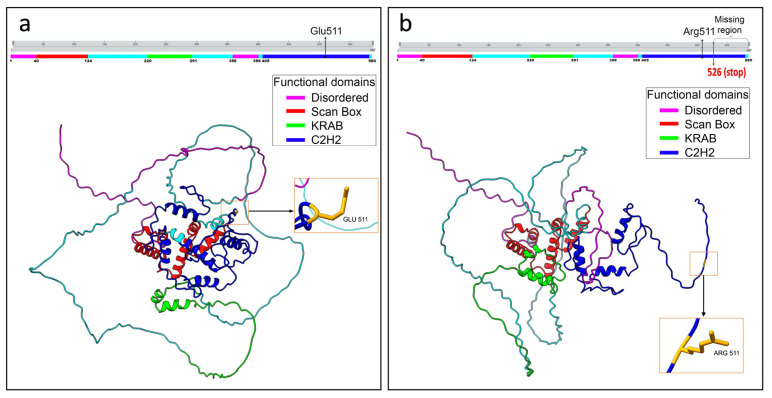
Graphical representation of the structural prediction of wild-type and mutated ZNF496 proteins. (**a**) Structural prediction of the wild-type protein. The panel placed above the model illustrates the functional domains in different colors. The black arrow in the panel indicates the wild-type residue Glu511, and the arrow on the protein structure points to the same residue. (**b**) Structural prediction of the mutated protein. The panel above the model illustrates the functional domains in different colors. The black arrow in the panel indicates the mutated residue Arg511, and the arrow on the protein structure points to the same residue. The curly bracket on the panel highlights the missing region of the protein, and the red arrow indicates the de novo stop site. Structural predictions were generated using UCSF ChimeraX software, version 1.8.

Structural analysis of the best selected models showed notable differences comparing both the wild-type and mutated ZNF496 best prediction models (Figure 3).

Specifically, the best-predicted models for the wild-type and mutated ZNF496 proteins accounted for 247 and 189 hydrogen bonds, respectively. The analysis revealed 93 hydrogen bonds present exclusively in the wild-type protein (Table S1). Notably, 59 of these were located downstream of residue Glu511, the specific site affected by the mutation. The analysis also unveiled 34 novel interactions that are present exclusively in the mutated protein (Table S2).

**Figure 4:**
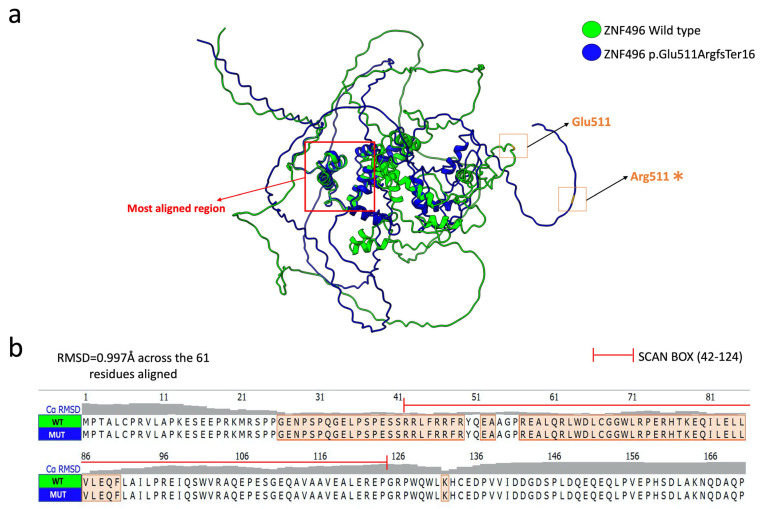
Structural alignment of the selected models of the ZNF496 protein wild-type (green) and mutated (blue). (**a**) Graphical representation of the superimposition of the two predicted models. The red arrow points to the main aligned region, while the wild-type residue Glu511 and the mutated residue Arg511 (*) are indicated by orange squares. The figure was generated using the structural prediction software UCSF ChimeraX. (**b**) Focus on the amino acid sequence spanning residues 1 to 170, highlighting the alignment of amino acid residues between the ZNF496 wild-type (WT) and mutated (MUT).

The structural superimposition of the two selected models showed an alignment of only 61 amino acids out of a total of 525, with a root mean square deviation (RMSD) value of 0.0997 Å. Figure 4 clearly illustrates the aligned residues, which are mainly located in the SCAN BOX domain.

**Figure 5:**
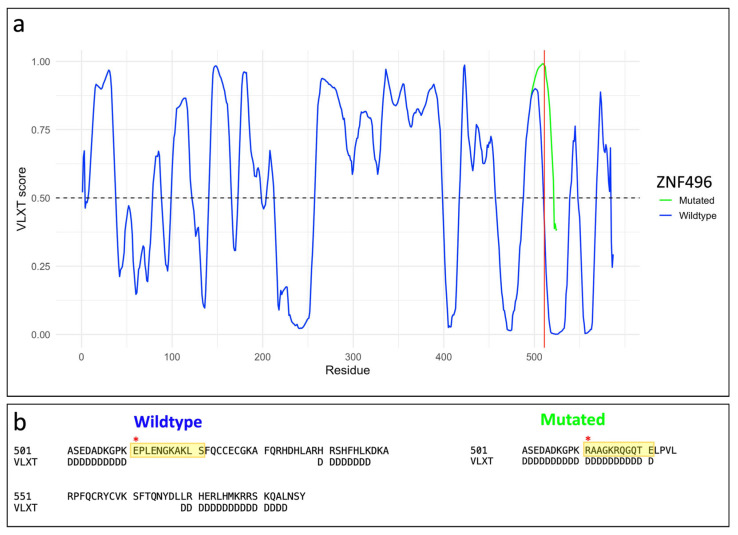
Structural disorder analysis of ZNF496 was performed using the PONDR software. (**a**) Variation of the VLXT score between the wild-type protein and the mutant carrying the p.Glu511ArgfsTer16 variant. Red line indicates the specific mutation site at position 511. The graph was generated in RStudio using the ggplot2 package. (**b**) Close-up of disorder variation (D) for each residue following position 511. As highlighted in yellow in the figure, due to the alteration of the polypeptide chain, the mutated protein shows a higher degree of disorder in the residues downstream of the mutation. The mutated motif in both the wild-type and mutated proteins is indicated by the red asterisk.

Furthermore, the protein disorder analysis conducted using PONDR revealed a structural alteration involving the 511–521 region, which is ordered in the wild-type protein but becomes disordered in the mutated form. Figure 5 illustrates the variation in the VLXT score, highlighting the differences in the degree of disorder between the two protein structures.

The predicted interaction models between ZNF496 and JARID2 revealed significant differences in the hydrogen bonding patterns between the wild-type and the mutated (p.Glu511ArgfsTer16) forms of ZNF496. Across the five predicted models for both the wild-type and mutated ZNF496–JARID2 complexes, the total number of hydrogen bonds averaged 1530.6 (±351.93) for the wild-type and 1099.4 (±366.24) for the mutated form.

Concerning the best selected models, the wild-type ZNF496-JARID2 complex exhibited a total of 1118 hydrogen bonds, whereas the mutated complex displayed only 947.

**Figure 6:**
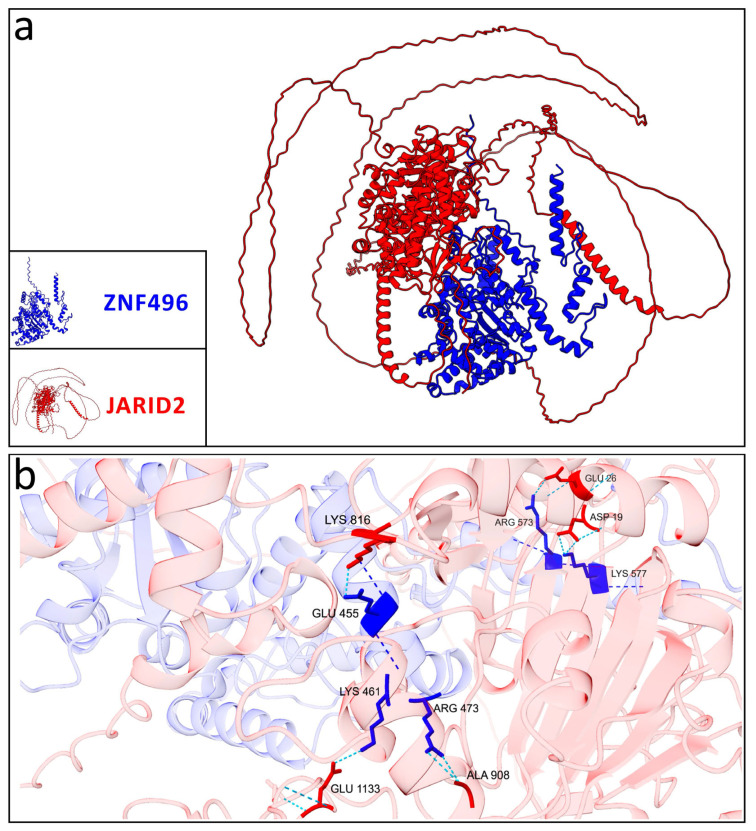
Graphical representation of the best model selected for the wild-type ZNF496-JARID2. (**a**) Interaction between the wild-type ZNF496 (blue) and JARID2 (red). (**b**) Close-up on the seven hydrogen bonds (turquoise dashed lines) listed in Table 2, formed between the wild-type ZNF496 and JARID2.

Additionally, the residues involved in these interactions differed significantly: the wild-type ZNF496 formed seven hydrogen bonds with JARID2 (Table 2; Figure 6).

**Figure 7:**
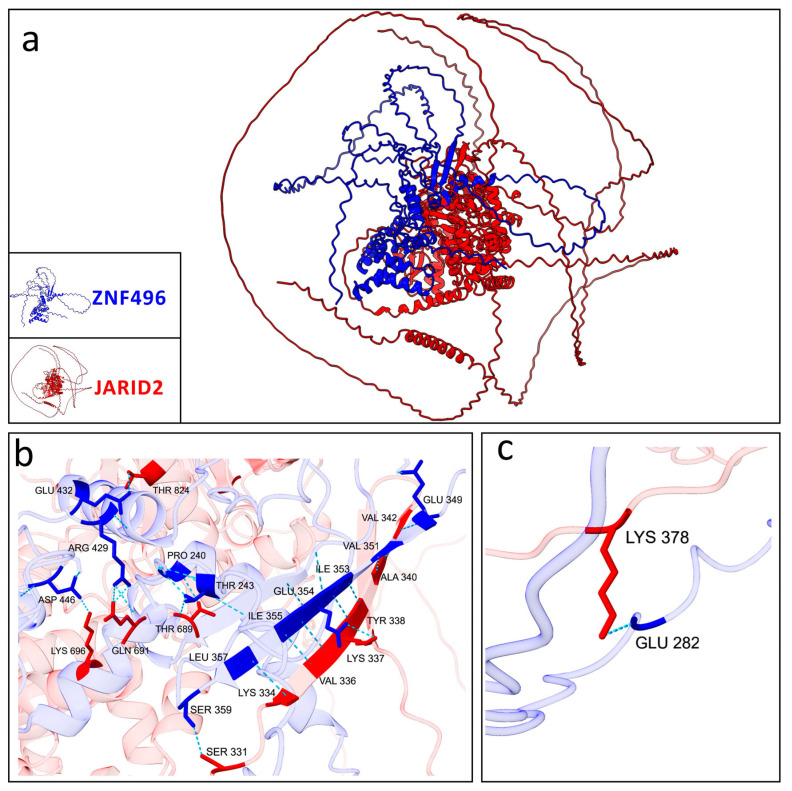
Graphical representation of the best model selected for the mutated ZNF496-JARID2. (**a**) Interaction between the mutated ZNF496 (blue) and JARID2 (red). (**b**,**c**) Close-up on the 18 hydrogen bonds (turquoise dashed lines) listed in Table 3, formed between the wild-type ZNF496 and JARID2.

Conversely, the mutated ZNF496 (p.Glu511ArgfsTer16) formed 18 hydrogen bonds interacting with JARID2 (Table 3; Figure 7).

## 3. Discussion

Over the years, NGS analysis has enabled the identification of novel genes associated with neurodevelopmental disorders. Among these, there are several genes belonging to the zinc finger superfamily [15,16].

In this study, WES analysis on the affected proband and both healthy parents revealed the presence of the de novo variant c.1530dup (p.Glu511ArgfsTer16), located in the *ZNF496* gene. Notably, no chromosomal abnormalities were detected by standard karyotyping or CGH-array analysis, nor were pathogenic variants found in known NDD-associated genes. Currently, the identified variant is absent from main variant annotation databases (e.g., HGMD, LOVD, dbSNP) and has no reported allele frequency in the aggregated population database gnomAD. The ClinVar database currently documents 63 missense single-nucleotide variants (SNVs) in *ZNF496*, with no reported frameshift variants. Of these, 61 SNVs (96.8%) are classified as variants of uncertain significance (VUS) with no associated phenotypic data. The remaining two variants (3.2%) are designated as likely benign. This variant spectrum highlights the complete absence of reported frameshift variants prior to our identification of p.Glu511ArgfsTer16. In fact, the variant identified in this study was submitted to the ClinVar database with the accession entry SCV006100880.

To date, no MIM phenotype number links *ZNF496* to a specific clinical phenotype. Thus, this study aims to propose *ZNF496* as a plausible candidate gene for NDDs. No prior studies have associated this gene with the patient’s clinical phenotype.

According to predictions by the DOMINO algorithm (score = 0.582), *ZNF496* exhibits an inheritance model intermediate between autosomal dominant and recessive. Since the variant is de novo (heterozygous in the proband and absent in the parents), we hypothesize an autosomal dominant mode of inheritance due to the de novo variant in a heterozygous condition.

**Figure 8:**
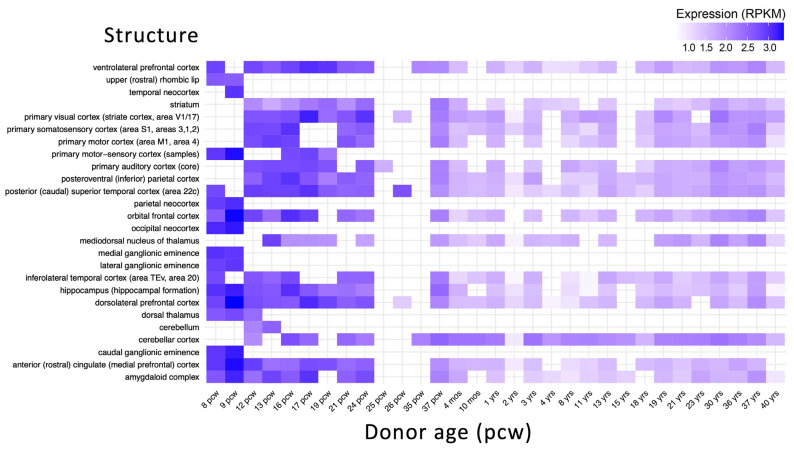
Developmental expression of *ZNF496* in the human brain. Expression levels are shown in reads per kilobase per million mapped reads (RPKM). The *x*-axis represents developmental stages from post-conceptional weeks (pcw) to years (yrs). The *y*-axis shows different brain structures. Data were obtained from the BrainSpan database. The heatmap was created using the ggplot2 and pheatmap packages in R Studio version 3.4.3.

Brain developmental gene expression data retrieved from BrainSpan database show that *ZNF496* is highly expressed between 8–24 post-conception weeks (pcw) in key regions, including the orbitofrontal cortex, dorsolateral prefrontal cortex, and primary motor/sensory cortices (Figure 8).

*ZNF496* encodes a C2H2-type zinc finger protein, a family frequently implicated in neurodevelopmental disorders [11]. The mutation, located in the transcript’s terminal exon, introduces a frameshift at amino acid 511, generating a premature stop codon at position 526. Despite the frameshift, NMDEscPredictor suggests the transcript escapes nonsense-mediated decay (NMD). This aligns with the “50-nt rule” (premature stop codons within −50 nucleotides of the last exon-exon junction often evade NMD) and the “last-exon rule” (stop codons in the final exon typically avoid degradation) [17,18]. Thus, the truncated protein is likely translated.

ConSurf analysis indicates that the region lost in p.Glu511ArgfsTer16 is highly evolutionarily conserved, potentially disrupting critical functions: protein binding (GO:0005515), DNA binding (GO:0003677), and transcription factor activity (GO:0000978; GO:0000981) within the nucleus (GO:0005634). The graphical output of the ConSurf analysis is shown in Supplementary Figure S1. The latter highlights the high solvent accessibility of the mutated residues, suggesting disrupted binding to DNA, RNA, or histone proteins. The mutation targets the C2H2 zinc finger domain, likely impairing DNA interaction. Moreover, AlphaFold3 structural predictions (Figure 3) reveal significant conformational changes in the mutant protein, driven by altered hydrogen bonding patterns. Such disruptions can destabilize tertiary structure [19]. PONDR analysis (Figure 5) further shows increased disorder in residues 511–521, implying reduced structural stability and supporting pathogenicity.

As documented, ZNF496 interacts with *JARID2*, a gene linked to developmental delay, intellectual disability, and facial dysmorphism (MIM phenotype entry number #620098). Physiologically, ZNF496 counteracts JARID2′s repressive activity on genes critical for neuronal differentiation [20], acting like a transcriptional activator. As predicted, the implicated domain involved in this interaction is the KRAB domain and the C2H2 domain [20]. However, it is important to note that the previously reported interaction between ZNF496 and *JARID2* [20] was identified using a two-hybrid screening approach, and this interaction has not been extensively characterized in other studies in the literature. The mutation is predicted to alter this interaction through deletion of ZNF496’s C2H2 domain, potentially leading to dysregulation of *JARID2* target genes. Computational modeling using AlphaFold3 revealed significant alterations in the bonding configuration of the mutated ZNF496-JARID2 complex compared to the wild-type complex. Specifically, we observed that the best prediction models selected an increase of 11 hydrogen bonds in the mutant complex compared to wild-type, and a complete rearrangement of all hydrogen bonding interactions. These findings suggested a fundamentally altered binding interface that may significantly impact the complex’s stability and functional activity. Nevertheless, all predicted models exhibited low reliability, as indicated by the low confidence scores in the predicted local distance difference test (pLDDT) values.

A key limitation of this study is the absence of Western blot analysis to confirm whether the mutation alters the molecular weight of the ZNF496 protein, confirming the production of the truncated protein. Additionally, we were unable to experimentally validate the predicted disruption of the ZNF496-JARID2 interaction. Future studies should address these gaps through biochemical assays to verify protein expression changes and characterize the functional consequences of this interaction loss.

In conclusion, our findings suggest *ZNF496* as a novel candidate for NDDs. However, undetected variants (e.g., deep intronic mutations or cryptic chromosomal imbalances) may contribute to the phenotype. Functional studies are needed to validate these hypotheses and elucidate the variant’s pathogenic role. To our knowledge, this is the first association in literature between *ZNF496* and NDDs.

## 4. Materials and Methods

### 4.1. Libraries Preparation and WES Analysis

Genomic DNA was obtained from peripheral blood leukocytes of the patient and both parents, according to a previously described method [21]. Specifically, a non-organic, non-enzymatic extraction method was used, which yields higher amounts of genomic DNA (approximately 300–500 ng/μL) [22]. Library preparation for the trio analysis and exome capture was carried out through the Agilent SureSelect V7 Kit (Santa Clara, CA, USA), according to the manufacturer’s protocol. Sequencing was performed using the Illumina HiSeq 3000 (San Diego, CA, USA), obtaining coverage of at least 20× for 97% of the targeted regions. Variant filtering was performed based on (i) presumed inheritance patterns—recessive, de novo, or X-linked—and (ii) a minor allele frequency (MAF) below 1%, referencing population databases including 1000 Genomes, ESP6500, ExAC, and gnomAD. The human reference genome assembly HG38 was used for variant alignment and analysis. All identified variants were subsequently validated by conventional Sanger sequencing, using the following primers: forward 5′-GGCCGGGCTTTGTCCTTCAGGTG-3′; reverse 5′-CCGCCTGAACTCCCACCTGCTCT-3′. The reaction was performed with the BigDye™ Terminator v1.1 Cycle Sequencing Kit (Life Technologies, Carlsbad, CA, USA) on the SeqStudio Genetic Analyzer (Thermo Fisher Scientific, Waltham, MA, USA).

### 4.2. Data Analysis

**Figure 9:**
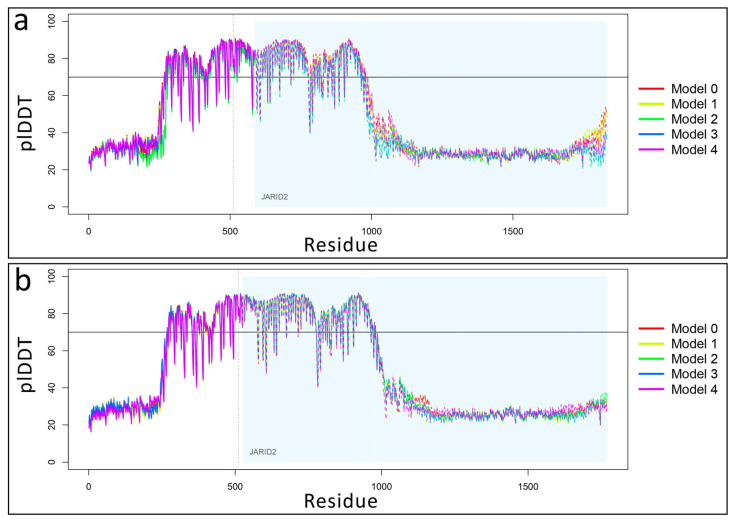
Variation of predicted local distance difference test (plDDT) values for ZNF496-JARID2 complexes. (**a**) Line plot showing plDDT score distribution across five predicted structural models of wild-type ZNF496 (with area) in complex with *JARID2* (light blue area). Model 0 was selected as “best model” for its highest plDDT average value of 53.073. Higher plDDT scores (range 0–100) indicate greater per-residue confidence in predicted atomic distances. (**b**) Line plot showing plDDT score distribution across five predicted structural models of mutated p.Glu511ArgfsTer16 ZNF496 in complex with *JARID2*. Model 2 was selected as “best model” for its highest plDDT average value of 52.132. The horizontal black line indicates a pLDDT value of 70, which was used as the threshold for confidence in the predicted structures. The dotted line separates the protein chains of ZNF496 and JARID2.

All the common variants, non-exonic polymorphisms, were excluded, filtering for the polymorphisms with a minor allele frequency (MAF) of <1% in the public databases gnomAD v.4.1.0, 1000 Genome Project, and Exome Sequencing Project (accessed on 11 June 2025). The pathogenic variants were searched on The Human Gene Mutation Database (HGMD Professional 2025.1) (accessed on 11 June 2025). Franklin by QIAGEN (Hilden, Germany) was used for filtering and prioritizing the genetic variants from the VCF file. The identified variant was classified using the “American College of Medical Genetics” (ACMG) guidelines and criteria [14]. The ClinVar database (https://www.ncbi.nlm.nih.gov/clinvar) (accessed on 25 June 2025) was used for submitting the genetic variant identified in this study. DOMINO [23] (https://domino.iob.ch/) (accessed on 11 June 2025) was used for predicting the inheritance pattern of the *ZNF469* gene, using a scoring system ranging from 0 (autosomal recessive) to 1 (autosomal dominant). BrainSpan database (https://www.brainspan.org/) (accessed on 11 June 2025) was used for retrieving the developmental brain expression data of a broad array of brain structures across the full course of human brain development. The expression data were elaborated using the packages pheatmap and ggplot2 of R Studio software version 3.4.3. Nonsense-mediated decay (NMD) escape prediction was performed using NMDEscPredictor (https://nmdprediction.shinyapps.io/nmdescpredictor/) (accessed 11 June 2025). Protein stability changes (ΔΔG) were calculated using MuPRO v.12.0 (http://mupro.proteomics.ics.uci.edu/) (accessed on 11 June 2025), with scores ranging from −1 (destabilizing) to +1 (stabilizing) [24]. Evolutionary conservation analysis was conducted via the ConSurf server (http://consurf.tau.ac.il/) (accessed 11 June 2025), which assigned conservation scores (1–4: variable; 5–6: intermediate; 7–9: conserved), functional annotations (functional/structural), and solvent accessibility (exposed/buried) for each residue. The PONDR server (https://pondr.com/cgi-bin/) (accessed on 11 June 2025) was used to predict disordered regions resulting from the mutation, based on the assignment of VLXT scores, which range from 0 (indicating structural order) to 1 (indicating structural disorder). Protein structures for both wild-type and mutated ZNF496 were predicted using AlphaFold3 (https://alphafoldserver.com/) (accessed 11 June 2025), with the highest-confidence model (based on plDDT score) selected from five generated predictions per variant. Protein modelling was performed using UCSF ChimeraX version 1.8. Protein–protein interaction models between ZNF496 (wild-type/mutant) and JARID2 were similarly generated and selected using the same criteria. Structural visualizations and model quality assessments were performed using R Studio v3.4.3 with bio3d, jsonlite, and ggplot2 packages, with comparative line plots generated for all predicted models (Figure 9).

## 5. Conclusions

In this study, we report a patient with GDD, mild ID, and DCD harboring the de novo variant c.1530dup (p.Glu511ArgfsTer16) in the *ZNF496* gene, identified through trio-based WES. Both parents were unaffected, and no other pathogenic variants in known neurodevelopmental genes were detected. The frameshift mutation alters residues 511–525, introducing a premature stop codon that truncates the protein from 525 to 587 amino acids—disrupting the functionally critical C2H2 zinc finger domain. Computational predictions suggest the mutated transcript escapes NMD.

In silico analyses revealed notable structural differences between the mutated and wild-type ZNF496 proteins, including altered interaction patterns with *JARID2*, suggesting potential dysfunction of this complex. To support the association of *ZNF496* with NDDs, we deposited this variant in ClinVar (SCV006100880). Functional studies are crucial for validating *ZNF496*’s role in NDDs and for characterizing the structural impact of this variant on ZNF496-JARID2 interactions.

## Figures and Tables

**Table 1:** ACMG criteria applied for the classification of the variant c.1530dup in *ZNF496* gene. The variant was classified as pathogenic following the ACMG guidelines [14].

Code	Criteria
PVS1	Null variant (nonsense, frameshift, canonical ± 1 or 2 splice sites, initiation codon, single or multiexon deletion) in a gene where LOF is a known mechanism of disease.
PM2	Absent from controls (or at extremely low frequency if recessive) in Exome Sequencing Project, 1000 Genomes Project, or Exome Aggregation Consortium.
PS2	De novo (both maternity and paternity confirmed) in a patient with the disease and no family history.

**Table 2:** List of the seven hydrogen bonds formed between the wild-type (WT) ZNF496 and JARID2 resulted from the AlphaFold3 prediction.

Protein A	Donor Residue	Donor Atom	Protein B	Acceptor Residue	Acceptor Atom	Distance (Å)
ZNF496 WT	Lys461	NZ	JARID2	Glu1133	OE2	3.413
ZNF496 WT	Arg473	NH1	JARID2	Ala908	O	3.289
ZNF496 WT	Arg473	NH2	JARID2	Ala908	O	3.309
ZNF496 WT	Arg573	NH2	JARID2	Glu26	OE1	3.266
ZNF496 WT	Lys577	NZ	JARID2	Asp19	OD1	2.661
ZNF496 WT	Lys577	NZ	JARID2	Asp19	OD2	2.849
JARID2	Lys816	NZ	ZNF496 WT	Glu455	OE2	2.769

**Table 3:** List of the 18 hydrogen bonds formed between the mutated (MUT) p.Glu511ArgfsTer16 ZNF496 and JARID2 resulted from the AlphaFold3 prediction.

Protein A	Donor Residue	Donor Atom	Protein B	Acceptor Residue	Acceptor Atom	Distance (Å)
ZNF496 MUT	Thr243	OG1	JARID2	Thr689	O	2.389
ZNF496 MUT	Val351	N	JARID2	Ala340	O	2.803
ZNF496 MUT	Ile353	N	JARID2	Tyr338	O	2.787
ZNF496 MUT	Ile355	N	JARID2	Val336	O	3.153
ZNF496 MUT	Leu357	N	JARID2	Lys334	O	3.453
ZNF496 MUT	Ser359	OG	JARID2	Ser331	OG	3.208
ZNF496 MUT	Arg429	NH1	JARID2	Gln691	OE1	2.492
ZNF496 MUT	Arg429	NH2	JARID2	Gln691	OE1	2.448
JARID2	Ser331	OG	ZNF496 MUT	Ser359	OG	3.208
JARID2	Val336	N	ZNF496 MUT	Ile355	O	3.172
JARID2	Lys337	NZ	ZNF496 MUT	Glu354	OE2	3.098
JARID2	Tyr338	N	ZNF496 MUT	Ile353	O	3.069
JARID2	Ala340	N	ZNF496 MUT	Val351	O	2.705
JARID2	Val342	N	ZNF496 MUT	Glu349	O	3.355
JARID2	Lys378	NZ	ZNF496 MUT	Glu282	O	2.897
JARID2	Thr689	OG1	ZNF496 MUT	Pro240	O	3.048
JARID2	Lys696	NZ	ZNF496 MUT	Asp446	OD2	2.063
JARID2	Thr824	OG1	ZNF496 MUT	Glu432	OE1	3.037

## Data Availability

The data presented in this study are available upon request from the corresponding author.

## Supplementary Materials

The supporting information can be downloaded at: https://www.mdpi.com/article/10.3390/ijms26157586/s1.

## Abbreviations

The following abbreviations are used in this manuscript:

ACMG	American College of Medical Genetics and Genomics
DCD	Developmental coordination disorder
GDD	Global developmental delay
ID	Intellectual disability
NDD	Neurodevelopmental disorder
NGS	Next-generation sequencing
NMD	Nonsense-mediated decay
IGV	Integrated genomics viewer
MAF	Minor allele frequency
plDDT	Predicted local distance difference test
SNV	Single-nucleotide variant
WES	Whole exome sequencing
