# Supplementary figures and images for: ZNF496 as Candidate Gene for Neurodevelopmental Disorders: Identification of a Pathogenic De Novo Frameshift Variant
Source: Int J Mol Sci. 2025 Aug 5;26(15):7586. doi: 10.3390/ijms26157586 (PMC12347329; doi:10.3390/ijms26157586)

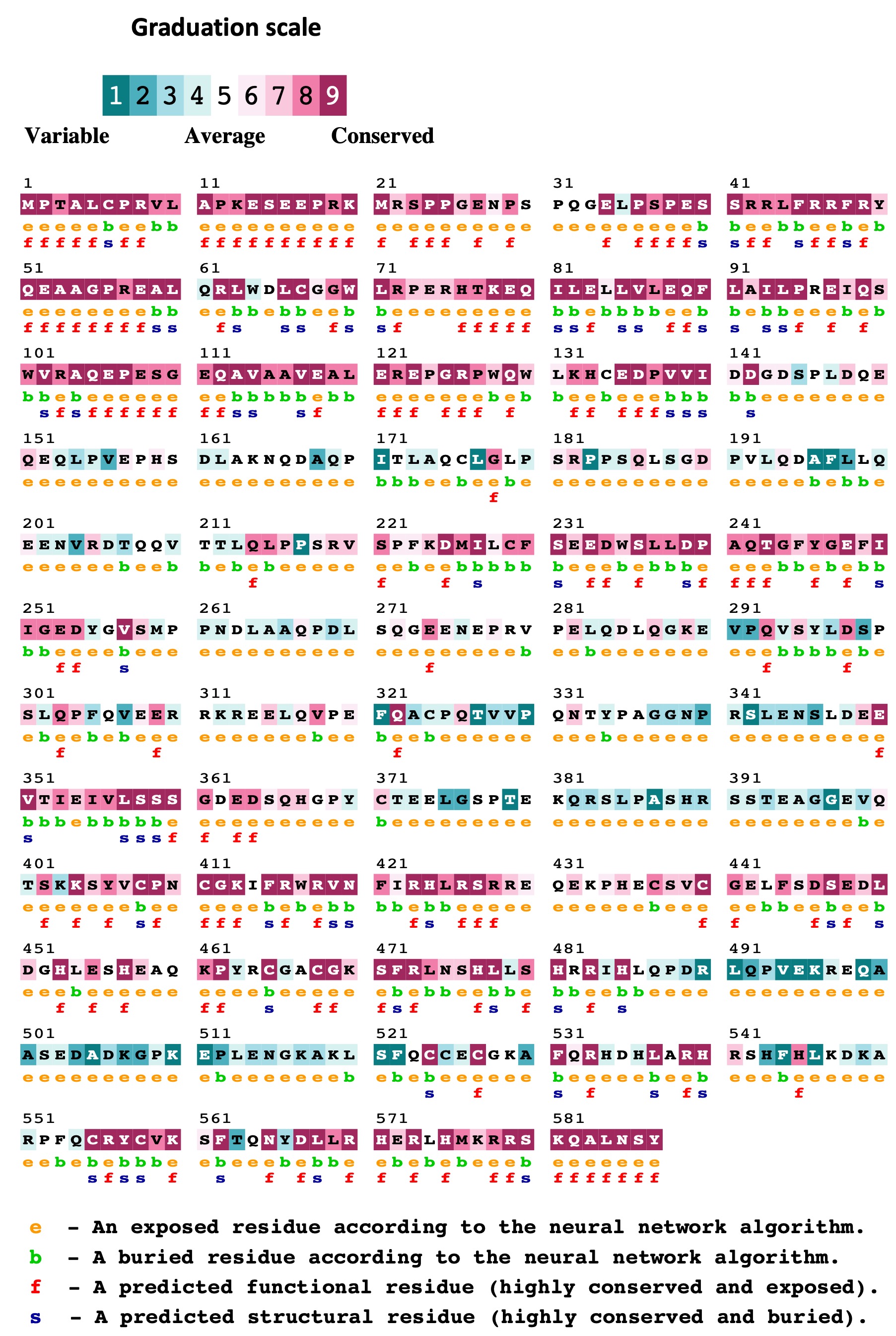

Supplement: Supplementary file 1 [file ijms-26-07586-s001.zip › Figure S1. ConSurf analysis of ZNF496 protein.jpg]
